# Non-contrast ultrasound image analysis for spatial and temporal distribution of blood flow after spinal cord injury

**DOI:** 10.1038/s41598-024-51281-7

**Published:** 2024-01-06

**Authors:** Denis Routkevitch, Zoe Soulé, Nicholas Kats, Emily Baca, Andrew M. Hersh, Kelley M. Kempski-Leadingham, Arjun K. Menta, Meghana Bhimreddy, Kelly Jiang, A. Daniel Davidar, Constantin Smit, Nicholas Theodore, Nitish V. Thakor, Amir Manbachi

**Affiliations:** 1 Department of Biomedical Engineering, Johns Hopkins University, Baltimore, MD USA; 2 Department of Neurosurgery, School of Medicine, Johns Hopkins University, Baltimore, MD USA; 3 HEPIUS Innovation Laboratory, School of Medicine, Johns Hopkins University, Baltimore, MD USA; 4 Department of Electrical and Computer Engineering, Johns Hopkins University, Baltimore, MD USA; 5 Department of Mechanical Engineering, Johns Hopkins University, Baltimore, MD USA; 6 Department of Anesthesiology and Critical Care Medicine, School of Medicine, Johns Hopkins University, Baltimore, MD USA

**Keywords:** Spinal cord diseases, Biomedical engineering, Ultrasound, Blood flow

## Abstract

Ultrasound technology can provide high-resolution imaging of blood flow following spinal cord injury (SCI). Blood flow imaging may improve critical care management of SCI, yet its duration is limited clinically by the amount of contrast agent injection required for high-resolution, continuous monitoring. In this study, we aim to establish non-contrast ultrasound as a clinically translatable imaging technique for spinal cord blood flow via comparison to contrast-based methods and by measuring the spatial distribution of blood flow after SCI. A rodent model of contusion SCI at the T12 spinal level was carried out using three different impact forces. We compared images of spinal cord blood flow taken using both non-contrast and contrast-enhanced ultrasound. Subsequently, we processed the images as a function of distance from injury, yielding the distribution of blood flow through space after SCI, and found the following. (1) Both non-contrast and contrast-enhanced imaging methods resulted in similar blood flow distributions (Spearman’s ρ = 0.55, p < 0.0001). (2) We found an area of decreased flow at the injury epicenter, or umbra (p < 0.0001). Unexpectedly, we found increased flow at the periphery, or penumbra (rostral, p < 0.05; caudal, p < 0.01), following SCI. However, distal flow remained unchanged, in what is presumably unaffected tissue. (3) Finally, tracking blood flow in the injury zones over time revealed interesting dynamic changes. After an initial decrease, blood flow in the penumbra increased during the first 10 min after injury, while blood flow in the umbra and distal tissue remained constant over time. These results demonstrate the viability of non-contrast ultrasound as a clinical monitoring tool. Furthermore, our surprising observations of increased flow in the injury periphery pose interesting new questions about how the spinal cord vasculature reacts to SCI, with potentially increased significance of the penumbra.

## Introduction

Traumatic spinal cord injury (SCI) significantly reduces patient quality of life. Following the initial impact, a secondary phase develops consisting of inflammation, swelling, and ischemia as the body responds to the primary insult^1–6^. This phase develops over approximately one week, and patient management during this first week is critical to mitigate the severity of damage. The current “gold-standard” is to elevate blood pressure with the aim of increasing blood flow to the site of injury^4,5^. However, this blood pressure augmentation is performed blindly, with no measure of effect on the injury site, and elevated blood pressure is associated with significant complications^1^. Spinal cord perfusion pressure, which incorporates blood pressure and intraspinal pressure, shows some improvement as a monitoring technique^7,8^, but this still does not fully inform the clinician on the actual status of injury.

Monitoring of spinal cord blood flow in the acute period shows great potential in improving our ability to treat acute SCI^9–11^. Several studies have measured blood flow in the spinal cord after injury and have noted an area of low to no perfusion at the umbra, or injury epicenter^12–17^. In these studies the penumbra, or periphery, has mostly been shown to have decreased perfusion compared to baseline, but with lesser magnitude change compared to the umbra. Some older studies, on the other hand, have noted some areas of increased blood flow after SCI, but it is unclear whether this is due to variability in injury severity, blood pressure, or measurement technique^18^. In human injuries, blood flow is more variable, and hyperperfusion has also been noted after SCI^19^. Despite attempts to establish patterns of blood flow after SCI, the importance of the different zones around the injury to patient neurological outcomes remains unclear. To better understand the significance of these zones, we set out to establish a technique for continuous blood flow monitoring after SCI. Eventually, we hope to apply this technique to clinical monitoring both intraoperatively and in the intensive care unit without restriction on recording length.

Ultrasound offers a potential solution to monitor blood flow in the spinal cord at the site of injury. Ultrasound imaging of the spinal cord is usually limited by attenuation of sound due to the vertebrae. However, the decompressive laminectomy performed after SCI removes the bony elements compressing the cord and allows for imaging^1,4^. Both contrast-enhanced and non-contrast ultrasound have been used to measure spinal cord blood flow. Non-contrast ultrasound uses Doppler-based methods to detect movement of blood within vessels^20^. Contrast-based methods rely on intravascular injection of microbubble contrast, which can be detected using contrast harmonic imaging modalities. The microbubble signal can be measured through time, and blood flow can be quantified through a variety of techniques^21–24^.

Within the research setting, contrast-enhanced ultrasound has been preferred over non-contrast ultrasound for the measurement of spinal cord blood flow. Traditionally, non-contrast ultrasound is only able to detect flow in blood vessels exceeding a certain size and flow velocity (i.e., arteries and arterioles) and misses small vessels with low flow velocities^20,25^. Consequently, non-contrast methods are unable to measure capillary perfusion, which represents how much blood is effectively delivered to the tissue^12^. Instead, non-contrast ultrasound can only measure flow through larger vessels within the imaging plane, only approximating capillary perfusion^20^. Contrast-enhanced ultrasound, on the other hand, can measure both large vessel flow and capillary perfusion.

This difference is particularly striking with 2-dimensional imaging, as capillaries and tissue within the imaging plane may receive blood from a larger vessel not in the plane. Thus, even if an imaging plane does not contain a large vessel, it still contains its branching capillaries, and the tissue receives blood flow. Even though neither technique would detect flow through the large vessel, contrast-based techniques could still detect perfusion through the in-plane capillaries it supplies. This would accurately reflect the amount of blood being delivered to tissue^26^. Non-contrast methods, however, would miss the large out-of-plane vessel. Without the ability to detect capillaries, non-contrast ultrasound would result in a deceptively low measure of blood flow despite adequate capillary perfusion. Given these challenges, non-contrast ultrasound has been underutilized, and further exploration of its strengths is warranted.

**Figure 1:**
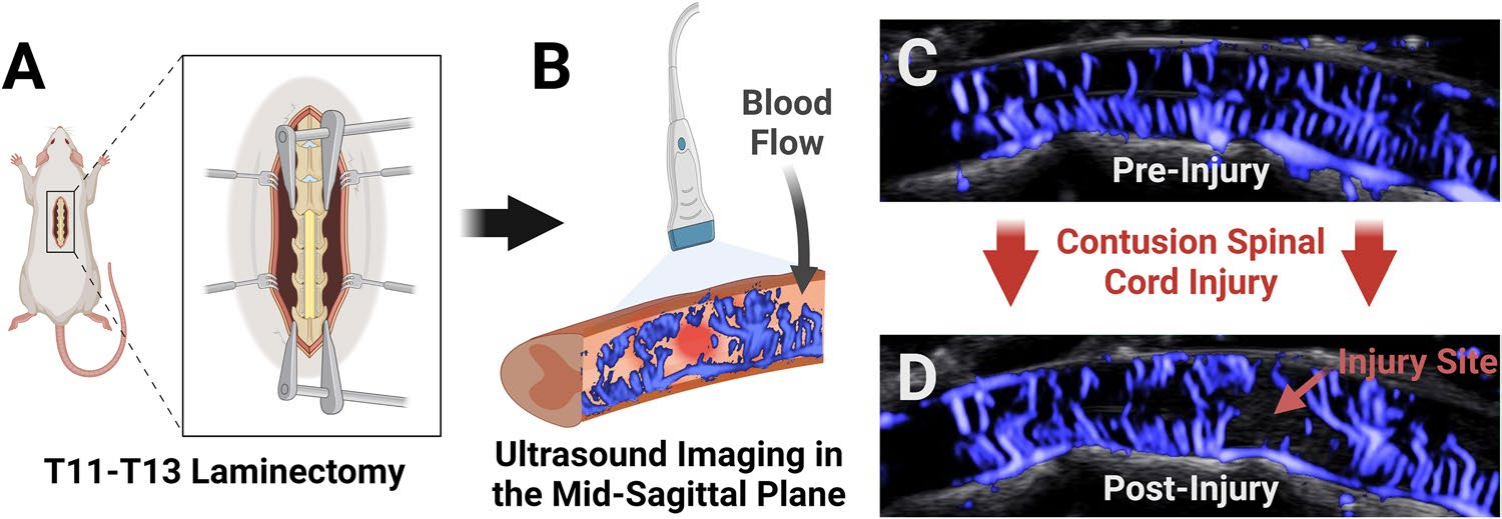
Overall diagram of experimental procedure. (**A**) A T11–T13 laminectomy was performed on a rat. (**B**) Ultrasound videos were taken in the mid-sagittal plane of the spinal cord, yielding blood flow images (**C**) before injury, and (**D**) after injury. Created using Biorender.com.

Contrast-enhanced imaging outside of the research setting, however, does have its own limitations. The clinical use of contrast-based techniques for patient monitoring is limited by the need for intravenous infusion of microbubbles. Although most studies have shown microbubble contrast to be relatively safe, adverse events can occur, such as allergic reactions and anaphylaxis^27–29^. Furthermore, microbubbles are usually administered via bolus dosing, whereas continuous infusion is a key requirement for long-term monitoring of patients with SCI. Although contrast agents may be diluted to allow for longer duration of monitoring, total monitoring time is still limited by a maximum allowed infusion amount. Non-contrast ultrasound does not require any infusion, and therefore there is no limit on monitoring duration. Moreover, new non-contrast techniques, such as Superb Microvascular Imaging (SMI)^25^ and functional ultrasound^30^, in combination with high-frequency ultrasound transducers, have overcome the barrier of measuring flow in small vessels with much lower flow velocity than was previously possible. Additionally, the anatomy of the spinal cord is particularly suitable for non-contrast imaging. The main perfusing vessels are the anterior spinal artery and its off-shooting sulcal arteries^31,32^. All these vessels lie within the mid-sagittal plane of the cord and can be captured using a single, carefully positioned imaging plane (Fig. 1B)^33,34^, reducing the potential effects from out-of-plane vessels mentioned above.

Therefore, to maximize the clinical impact of ultrasound-based monitoring of spinal cord perfusion, we seek to establish non-contrast ultrasound as a translatable blood flow imaging modality for patients with SCI. To accomplish this, we will first demonstrate that a monotonic relationship exists between flow velocity and our non-contrast ultrasound measures in a benchtop experiment. Then, we will show functional similarity of non-contrast ultrasound to contrast methods in the spinal cord by comparing post-injury images taken with each modality in the same imaging plane. We expect there to be a good correlation between non-contrast and contrast methods. As a result, once validated, non-contrast methods will present a viable alternative for blood flow measurement in SCI. Furthermore, we hope to further validate non-contrast imaging by showing similar blood flow patterns after SCI to the reported literature, as described above. To accomplish this, we will analyze ultrasound images obtained without contrast and demonstrate the spatial extent of injury at different severities. This will be the first analysis of non-contrast ultrasound blood flow recordings of the spine to this resolution and may reveal interesting new blood flow patterns. Finally, we will demonstrate the feasibility of non-contrast ultrasound for long-term, continuous blood flow measurement with high temporal resolution by monitoring injury regions in the hyperacute period. Since the length of this recording is not restricted by contrast, the demonstration of the ability to detect blood flow dynamics even for short duration will serve as proof-of-concept for long-term monitoring. Taken together, we hope this study will establish the validity of non-contrast ultrasound and enable further development of this technique in various clinically relevant scenarios, especially for directing the acute care management of spinal cord injury.

## Materials and methods

### Animals

All animal experiments were conducted in compliance with the National Institutes of Health guide for the care and use of laboratory animals (NIH Publications No. 8023, revised 1978). The experimental protocol was approved by the Johns Hopkins University Animal Care and Use Committee. All methods were performed following the ARRIVE guidelines (Animal Research: Reporting of In Vivo Experiments). A total of 34 female Sprague–Dawley rats (11 weeks, Charles River Laboratories, Wilmington, MA, USA) were used for this study. The choice of sex and age were based on standard models found in the literature^35^.

It should also be noted that this study was conducted purely on female rats. Our group uses this model as the post-operative bladder expression is more easily performed in female rats. Although the literature shows minimal differences in functional outcomes between male and female rats^36,37^, further exploration of effects of sex on spinal cord blood flow after injury is warranted. This is especially true as this technique is applied in clinical settings, where spinal cord injury pathophysiology could be more dependent on patient sex.

All rats underwent a T11–T13 laminectomy to expose the spinal cord. For the injury severity experiment, 24 rats underwent recordings before injury and at 15 min post-injury using 5-s video recordings to capture multiple cardiac cycles. The 15-min wait period was chosen to allow the blood flow to stabilize before commencing recording. They received injuries of 100 kDyn (mild, n = 10), 175 kDyn (moderate, n = 5), or 250 kDyn (severe, n = 9). To determine if probe removal and replacement influenced measurements, 5 additional rats did not undergo an injury but underwent imaging pre- and post-probe replacement. Seven of the injury severity rats additionally underwent contrast harmonic imaging with contrast injection for the contrast comparison experiment (see section “Contrast-enhanced recording”). Continuous monitoring was performed on five additional rats as follows. In this experiment, pre- and post-injury recordings were performed continuously for 15 min. This length of time was chosen to demonstrate proof-of-concept of continuous recording using non-contrast ultrasound, and to elucidate the blood flow dynamics that occur in different regions immediately after injury. Rats that underwent continuous monitoring were all injured at 250 kDyn (severe). This information is summarized in Supplementary Table S1.

### Laminectomy and spinal cord impact

After induction of anesthesia with 3% isoflurane in room air, rats were maintained at 1.8–2% isoflurane. The inhaled concentration was titrated based on animal physiologic monitoring, including through respiratory rate and paw-pinch reflex. The T11–T13 vertebrae (Fig. 1A) were identified by palpation of the last floating rib. These levels were chosen due to their large size, enabling a three-level laminectomy to span the maximal possible tissue, maximizing the spatial distribution available for hemodynamic study. An incision was made above the chosen vertebrae, and the paraspinal muscles were cut away from the T10–L1 vertebrae. The T10 and L1 spinous processes were clamped using a stereotaxic frame with spine clamps (David Kopf Instruments, Tujunga, CA, USA). Light traction was applied to position the spine with minimal lateral curvature. Bone cutters^17^ were used to cut the lamina and expose the spinal cord. After pre-injury imaging, the spinal cord was contused using an Infinite Horizons IH-0400 spinal cord impactor (PSI Impactors, Fairfax Station, VA, USA) at the level of T12.

### Microfluidic validation of non-contrast measures

The non-contrast measures were validated using a benchtop flow model, or phantom^33,38^. The phantom was constructed using polydimethylsiloxane (PDMS) with suspended copper wiring in two different diameters (408 µm and 506 µm), as those sizes were commercially available. PDMS was chosen due to ease of manufacture and durability, but its low sonolucency prevents measurement of flow through smaller diameters. After curing the polymer, the wiring could be removed to create a vessel-like structure. The phantom was submerged in water and the ultrasound probe was positioned above it (Supplementary Fig. S1A).

Doppler fluid (CIRS, Norfolk, VA, USA) was injected into the phantom at precise flow rates using a syringe pump and images were acquired for 15 s at various SMI scales. In the Canon system, “scale” is a value related to the pulse repetition frequency that modifies the sensitivity of the system to low-flow signals as well as noise. Scale values chosen were 0.4 (high sensitivity), 0.9 (intermediate sensitivity), and maximum scale (low sensitivity) which is determined by the machine at time of recording. Area-adjusted velocity index, as described below, was extracted from the recordings and averaged over the entire recording period. This parameter could then be compared to the known flow velocity, calculated from flow rate and phantom cross-sectional area.

### Non-contrast imaging

Imaging was performed using an Aplio i800 ultrasound machine with an i22LH8 probe (Canon USA, Melville, USA). Blood flow was visualized using SMI (Doppler transmit center frequency 12 MHz, 39 fps, maximum scale), due to the ability to accurately detect low flow vessels. Although this 39 Hz (fps) sampling frequency is below the ultrahigh sampling rates used in contrast-based techniques, it is well above the maximum frequency of 0.5 Hz relevant in clinical monitoring^39–41^. The probe was positioned over the midsagittal plane of the spinal cord (Fig. 1B) using a 3D-printed probe holder, a ball-and-socket joint, and the stereotaxic frame. Small translational and rotational adjustments were made until the vasculature through the entire length of the exposed spinal cord was clearly visible. Pre-injury imaging was performed (Fig. 1C) and the stereotaxic coordinates were noted. The stereotaxic arm was rotated away from the cord during impact and returned to the exact same coordinates for post-injury imaging (Fig. 1D). Velocity index maps were extracted from the images using a color-to-velocity-index transform^42^. For the contrast comparison and injury severity experiments, each 5-s duration velocity index map was averaged over time to obtain a single image for each pre- and post-injury recording.

### Contrast-enhanced recording

After performing the non-contrast imaging described above, rats allocated for contrast-based imaging underwent injection of a 400 µL bolus of Lumason microbubble contrast (Bracco, Milan, Italy), followed by a 0.5 mL saline flush, through a tail vein catheter. The spinal cord was recorded using contrast harmonic imaging for 150 s. This imaging was performed immediately after the SMI pre-injury recording with no change in the probe or spine position to enable accurate comparison of the modalities. After a 30-min washout, SCI was induced as before and recording with each modality was performed again. Time between contrast-enhanced and non-contrast recordings was 1–2 min, during which any change in blood flow was assumed to be minimal.

### Processing contrast-enhanced images

**Figure 2:**
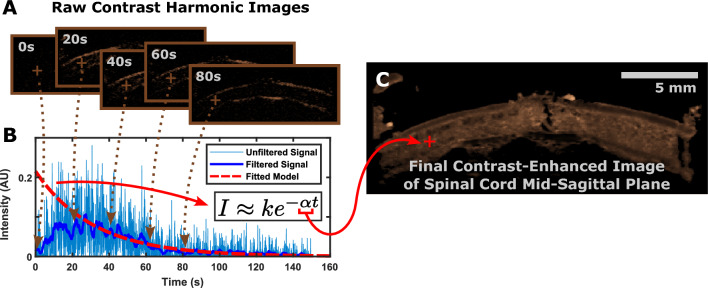
Method for obtaining contrast-enhanced images. (**A**) Sample frames from a contrast harmonic imaging video. (**B**) Intensity values of individual pixels were plotted over time, filtered through an averaging filter, and were used to fit a first order exponential decay function. (**C**) The resulting decay constant was used as the pixel’s value to construct the final image.

To generate the contrast-enhanced image (Fig. 2), the value of each pixel in the contrast harmonic recording (Fig. 2A) was plotted over time. The signal was processed using a simple moving average filter 32 samples in length for noise reduction. The filtered signal, starting from the time of maximum signal intensity, was then fit to a first order exponential decay model. The magnitude of the decay constant of the model (Fig. 2B) was used as the surrogate for flow with units of 1/s^23^. The higher the decay constant magnitude, the faster the contrast agent is cleared from the cord, indicating greater perfusion. This parameter was chosen as opposed to peak height or other parameters as it is more robust to error introduced by differences in the contrast bolus amount^21–23^. Certain pixels did not receive any microbubble signal and thus were assigned a value of 0 1/s. These pixels were chosen as those that did not reach a maximum intensity value of 0.015 AU or that did not achieve peak flow until 75 s into recording.

The resulting images contained speckle noise, potentially due to occasional aggregated microbubbles detected in the tissue, artificially increasing the peak intensity in the first-order model (Fig. 2B) and resulting in an elevated decay constant. Although hand-mixing is sufficient according to manufacturer instructions, we recommend frequent vortexing of the microbubbles to prevent aggregation prior to injection. To counteract this, the images were processed using a morphological opening operator with the MATLAB offset disk-shaped structuring element (5-pixel radius, 0.05 1/s height), yielding the final image (Fig. 2C).

### Pixelwise processing to obtain the spatial distribution

**Figure 3:**
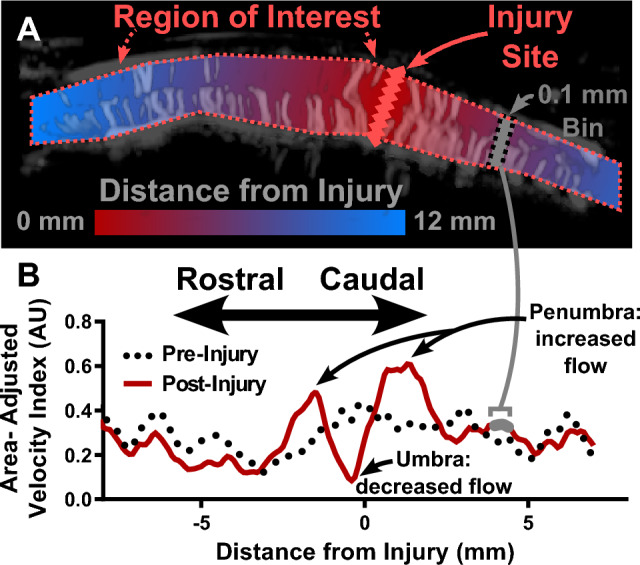
Obtaining a distribution of blood flow over distance from ultrasound images. (**A**) An injury marker and a region of interest including only the perforating vessels were drawn over the images. The distance of each pixel in the region of interest from the injury is shown via the color bar. (**B**) The post-injury spatial distribution curve (solid red) was constructed by binning pixels into distance bins 0.1 mm in size (not drawn to scale) and calculating the area-adjusted velocity index. Pre-injury spatial distribution in the same rat (dotted black) is also shown for comparison.

Both the contrast and non-contrast images were processed using pixelwise analysis to yield the spatial distribution. This analysis was inspired by Soubeyrand et al.^16^ with higher spatial resolution and the addition of distance as a numeric variable. This analysis resulted in ~ 200 regions of interest, compared to the 7 previously reported^16^. In this pipeline (Fig. 3A), injuries were labeled as a line perpendicular to the long axis of the spinal cord. Additionally, the upper and lower borders of the spinal cord were drawn manually to exclude any signal from the anterior and posterior spinal arteries. The minimum Euclidean distance was then calculated from each pixel in the images to the injury line. Pixels in the rostral portion of the image (left of injury) were chosen to have negative distance and pixels in the caudal portion (right of injury) were chosen to have positive distance.

Pixels were then binned according to distance into bins 0.1 mm wide and the average area-adjusted flow parameter was calculated for each image. The flow parameter was velocity index (arbitrary units, AU) for the SMI images and decay constant (1/s) for the contrast-enhanced images. The area-adjusted parameter (*I*, Eq. 1) was introduced by Scholbach and Scholbach^43^ and represents the product of average pixel brightness ($v$) with fractional area of colored pixels (${A}_{px}$) over total bin area (${A}_{ROI}$). We have found this parameter to be more sensitive to small changes in blood flow when compared to pixel brightness alone. It also includes normalization of the flow parameter to the bin area, which was necessary as not all distance bins had the same total number of pixels. The parameter was then filtered using an averaging filter 1 mm in width to improve inter-rat comparison and ameliorate small differences in position and spacing of individual vessels. Plotting this parameter against distance yielded the spatial distribution of blood flow (Fig. 3B).

1 $$I=v*\frac{{A}_{px} \,\left[c{m}^{2}\right]}{{A}_{ROI}\,\left[c{m}^{2}\right]}$$

### Labeling of injury zones

We used the output of the pixelwise analysis of non-contrast images to determine locations of different zones of injury. The MATLAB findpeaks function was used on the post-injury spatial distributions with the minimum peak prominence parameter set at 0.03 AU. This parameter was chosen after visually confirming accurate zone labeling in multiple representative rats, and then was held constant for the remainder of the study. The two closest peaks on either side of 0 mm distance were chosen as the rostral and caudal penumbras. The distance where minimum flow between the two peaks was observed was labeled as the injury epicenter, or umbra. If the minimum flow was detected at more than one consecutive distance bin, the center of the distance bins was used to label the epicenter. This epicenter value did not always match the manual injury label, perhaps due to image analysis revealing details not visible to the human eye. Distal (presumably unaffected) regions were then labeled as all points rostral to the rostral penumbra and caudal to the caudal penumbra. Single points were chosen for the umbra and penumbras to emphasize the differences in flow, but it may be advantageous in different applications to define these points as regions as well. Finally, penumbra prominence was defined as the difference in blood flow between each penumbra and the umbra.

Blood flow for each injury zone was then calculated as the average area-adjusted velocity index at the locations corresponding to that zone. Finally, we found the injury rostral-caudal extent as the distance between the two half-maximum points on either side of the umbra. These points are defined by the location at which the flow is equal to the average between the penumbra and umbra values. Pre-injury parameters (umbra and penumbra blood flow, penumbra prominence) were similarly determined as stated above from the pre-injury distribution using the locations from the post-injury distribution. Although the algorithm was kept constant after initial development, during all subsequent analyses, correct labeling of injury zones was verified through visual inspection of each trace with labeled zones.

### Flow over time

To obtain recordings of blood flow over time, the extended-length (15 min pre-injury, 15 min post-injury) continuous recordings were obtained as described above (see section “Animals”). Instead of taking the average velocity index map over time, they were analyzed frame-by-frame using the analysis described above (see sections “Pixelwise processing to obtain the spatial distribution” and “Labeling of injury zones”). The locations of the umbra and penumbra occasionally had jump discontinuities in time due to errors in their labeled position. Since biologically, the injury regions would not be expected to exhibit these types of discontinuities, their locations were median filtered with a filter size of 40 frames. Each flow parameter could then be plotted against time to see its evolution.

### Statistical analysis

GraphPad Prism version 9.5.1 and MATLAB R2022b were used for all statistical analyses and plots. To determine the correlation between contrast-enhanced and non-contrast ultrasound, Spearman’s correlation coefficient (ρ) was calculated for the values of each modality at each distance bin. Spearman’s coefficient was chosen to show only monotonicity and avoid any assumptions of linearity between the contrast-enhanced and non-contrast methods. Average correlation among multiple rats was calculated by using Fisher’s *z* transformation^44,45^. This method of averaging correlations was chosen over a single, multi-rat correlation as there was greater inter-rat variability between the contrast-enhanced methods than non-contrast. This error likely arose from variations in the small volume (400 µL) injection of concentrated microbubbles. Least squares spline models (Shape Language Modeling Toolbox for MATLAB) were fit to this data for visualization^46^. This model was chosen to avoid assumptions about linearity or specifics of the monotonic relationship between contrast-enhanced and non-contrast methods. To determine effects of injury severity, a two-way repeated measures analysis of variance (ANOVA) was used with factors of pre- vs post-injury timepoints and injury severity. If the ANOVA detected a statistically significant difference, the Šidák multiple comparisons test was used to determine differences between injury severity groups. Significance was set at p < 0.05.

## Results

### Benchtop validation of velocity index

**Figure 4:**
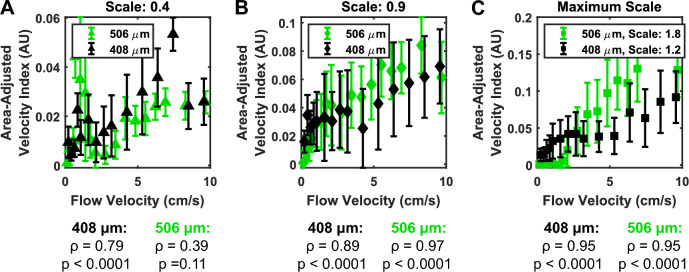
Benchtop ultrasound flow experiments demonstrated monotonic relationship between flow velocity and area-adjusted velocity index. The theoretical relationship between area-adjusted velocity index and flow velocity was probed by passing Doppler fluid through polydimethylsiloxane microfluidic flow phantoms at various flow rates. (**A**) Low scale (high sensitivity) showed susceptibility to noise, especially at lower flow velocity, which interfered with the monotonic relationship. At higher flow rates, this improved somewhat, but Spearman’s correlation remained low. This susceptibility to noise is undesirable, and thus we have avoided low scale recordings. (**B**) At scale of 0.9 (intermediate sensitivity) and (**C**) at maximum scale (minimum sensitivity), the monotonic relationship between velocity and flow is clear. Both scales resulted in highly statistically significant Spearman’s rank correlation, confirming monotonicity of the velocity-to-velocity-index relationship.

First, we validated the use of velocity index as a surrogate for velocity through an ultrasound flow phantom, or model. Figure 4 shows the results of the benchtop validation experiment. Spearman’s correlation was used to determine monotonicity but not linearity between velocity and velocity index. At the low scale parameters (high sensitivity), the correlation was significant only for the 408 µm size (Fig. 4A). At higher scale (lower sensitivity), the correlation was much improved, with the correlation ≥ 0.95 in both sizes occurring at maximum scale, which is set by the machine (Fig. 4C). A diagram of the experimental setup and example images can be found in Supplementary Fig. S1.

### Similarity of the non-contrast distribution to contrast-enhanced ultrasound

**Figure 5:**
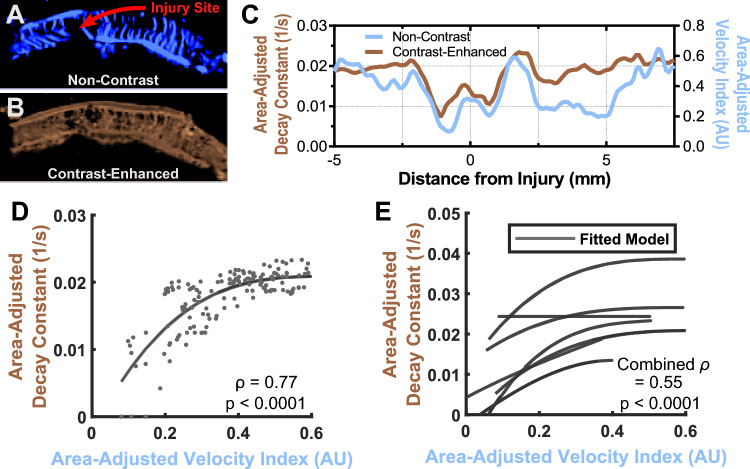
Spatial distribution of spinal cord blood flow is similar in non-contrast and contrast-enhanced ultrasound. (**A**) Non-contrast and (**B**) contrast-enhanced images were taken with the probe in a constant position above the spinal cord. (**C**) Each image was processed into its corresponding spatial distribution curve, with the representative curve showing good agreement between the two modalities. (**D**) Plotting the non-contrast value of each distance bin against its associated contrast-enhanced value resulted in a Spearman’s correlation of 0.77 (p < 0.0001) in this representative rat. (**E**) This correlation held across multiple rats, shown here fitted to a least squares spline model for visualization, with an average correlation of 0.55 (p < 0.0001) as calculated using Fisher’s *z* transformation. A single rat had poor correlation, perhaps due to out-of-plane positioning of the ultrasound probe disproportionately affecting the non-contrast image.

Next, we set out to determine the validity of non-contrast ultrasound measurements in vivo. As mentioned before, non-contrast measurements can capture flow through in-plane vessels, but may miss perfusion of tissue from out-of-plane vessels. Therefore, non-contrast (Fig. 5A) and contrast-enhanced images (Fig. 5B) were taken in seven rats in the same imaging plane, both before and after injury. Each image was processed using the technique detailed in Fig. 3 to yield the distributions in Fig. 5C. The resulting blood flow distributions were then compared as a function of distance. In Fig. 5D, an example plot between the non-contrast and contrast-enhanced blood flow measures shows correlation, though not necessarily linearity, between the two measures (Spearman’s ρ = 0.77, p < 0.0001). When compared among different rats, the estimated monotonic relationship between the two changed. Overall, the correlation was maintained when averaged among all the rats (Fig. 5E, ρ = 0.55, p < 0.0001). One rat in Fig. 5E showed no significant correlation between the two modalities. Excluding this rat, average correlation increased to ρ = 0.67, p < 0.0001. Error between the two modalities as a function of distance from injury can be found in Supplementary Fig. S2.

### Interpretable blood flow parameters

We next show that non-contrast ultrasound can detect blood flow changes after SCI. Figure 3B shows a representative distribution of blood flow 15 min after spinal cord injury, with corresponding pre-injury flow also shown. At the epicenter of the injury, when compared to pre-injury, there was a relative paucity of blood flow. As the distance from injury increased in both directions, blood flow increased to a level above baseline in tissue that was affected by injury but not completely destroyed. Distal to the injury, the blood flow returned to pre-injury values in presumably unaffected tissue.

**Figure 6:**
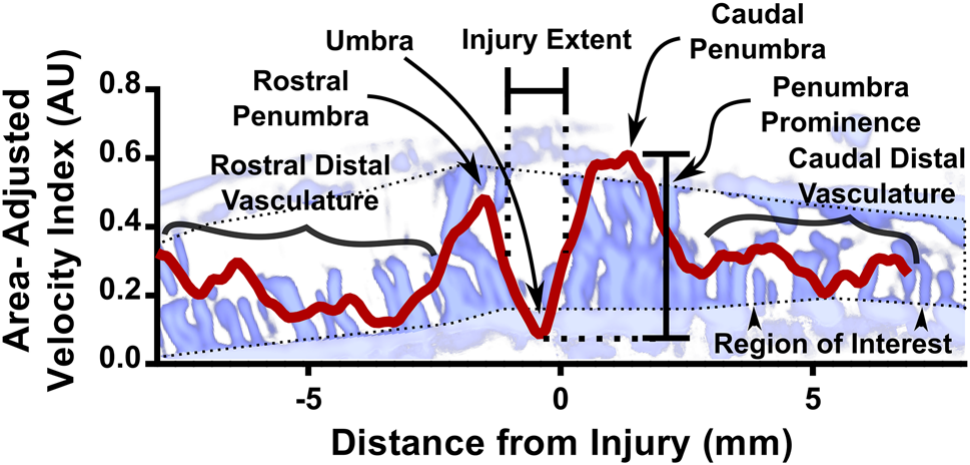
Parameters that can be extracted from the distance distributions shown in a representative distribution 15 min after SCI. The minimal flow value of the distribution was labeled as umbra flow. Due to human error in drawing the injury location, this did not always occur at zero distance. The peaks on either side of the umbra were termed the penumbra. Penumbra parameters included their raw index values as well as the difference from the umbra (prominence). Injury extent was calculated as the distance between the two points occurring halfway between the umbra and each penumbra. Distal, presumably healthy, distance bins are also shown.

For simpler analysis, we extracted a set of interpretable parameters from the distribution (Fig. 6). Based on the shape of the distribution, we measured the blood flow at the umbra and both the rostral and caudal penumbras (see sections “Pixelwise processing to obtain the spatial distribution” and “Labeling of injury zones”). Additionally, we measured the average blood flow of the distal tissue in each direction. We then also measured the prominence of each penumbra as shown in Fig. 6, which was calculated as the difference in blood flow between the penumbra peak and umbra trough. Since the injury site was initially manually labeled to establish a reference for the spatial distribution, the umbra location was not always at 0 mm. The average penumbra location was at -0.24 mm ± 0.61 mm, which was not statistically significant compared to 0 mm.

### Parameters pre- and post-injury and dependence on injury force

**Figure 7:**
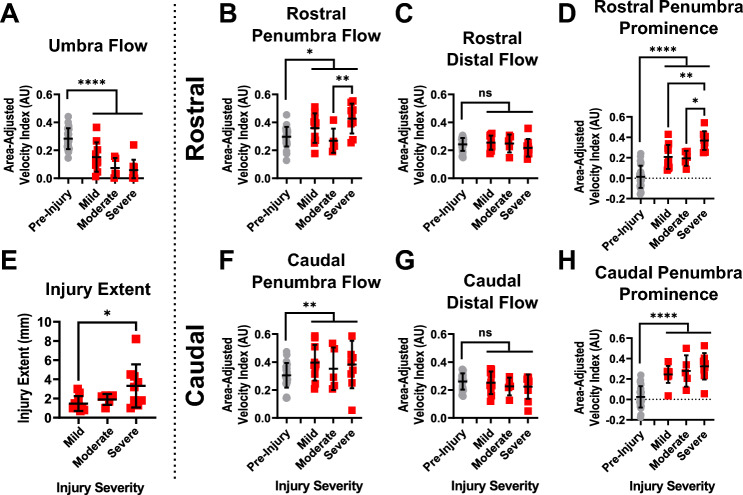
Spatial distribution parameters show strong ability to detect spinal cord injury and more limited ability to detect injury severity. (**A**) Umbra flow showed significant decrease from pre- to post-injury, and trends of decreasing flow as severity increased. (**E**) Injury extent showed significant increase from the mild to severe injuries. (**B**,**F**) Flow in the penumbra, both rostrally (**B**) and caudally (**F**), was significantly elevated as compared to pre-injury values. However, only the rostral penumbra had significant differences between injury types. (**C**,**G**) The distal flow was unchanged from pre-to post-injury in both directions. (**D**,**H**) The penumbra prominence showed greatest separation between both pre- and post-injury comparisons and between injury severities. Again, however, only the rostral penumbra (**D**) showed significant differences between injury types. Significance of differences between pre- to post-injury reported from two-way repeated measures ANOVA. Significance of differences between injury severities calculated using Šidák multiple comparisons. *p < 0.05, **p < 0.01, ***p < 0.001, ****p < 0.0001, *ns* not statistically significant.

We next aimed to use these parameters to quantify blood flow changes 15 min after spinal cord injury. The first comparison to this effect was the pre- to post-injury difference in the parameters. The second question we hoped to answer was how these changes are further affected by severity of the injury. Figure 7 summarizes the findings. It should be noted that the comparison from pre- to post-injury was performed pairwise, as each measurement was performed on each rat. The comparison between injury severities, however, was not pairwise, as different rats were injured at different impact forces. To confirm that probe replacement was not a factor, we also measured each parameter before and after probe replacement without injury (Supplementary Fig. S3). The data showed no statistically significant difference in any of the parameters due to probe replacement.

Flow at the site of the umbra was significantly decreased post-injury when compared to pre-injury (Fig. 7A). This is expected based on our qualitative analysis above (see section “Interpretable blood flow parameters”). The umbra flow trended towards decreasing with respect to increasing injury severity, although this effect did not achieve statistical significance. Conversely, the injury extent increased with respect to severity, achieving significance in the comparison between mild and severe injuries (Fig. 7E).

The penumbras both rostrally and caudally showed increased flow after injury compared to baseline (Fig. 7B,F). The rostral penumbra in the severe injury case also showed a significantly higher flow than in the moderate injury case only. The caudal penumbra, however, showed no difference between the injury severities. As expected, this change was not reflected in the distal flow in each location, as neither the rostral nor caudal distal tissue showed any change from pre- to post-injury (Fig. 7C,G).

The final parameter analyzed was the prominence of each penumbra, or the difference between the penumbra and umbra flow (Fig. 7D,H). This parameter showed the greatest difference between pre- and post-injury groups in both the rostral and caudal cases. Furthermore, in the rostral penumbra, there was a significant difference between the severe injury group and both the mild and moderate groups. Although the trend was not significant in the caudal group, the mean prominence slightly increased from mild to moderate and moderate to severe.

### Tracking of regions through time

**Figure 8:**
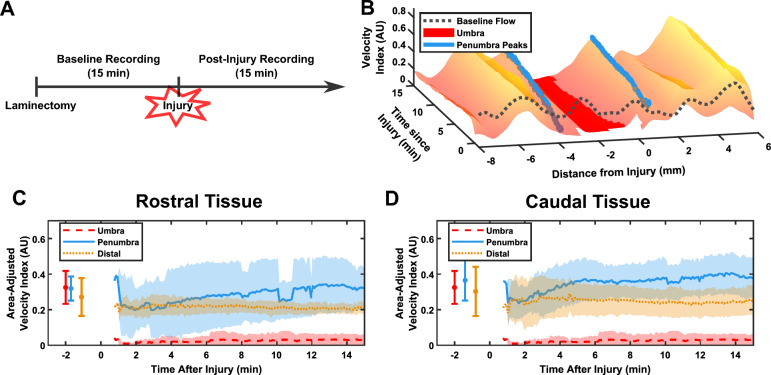
Non-contrast ultrasound shows new blood flow dynamics in the penumbra. (**A**) Experimental timeline showing the continuous recording of 15 min pre- and post-injury. (**B**) Surface plot showing the change of the spatial distribution of blood flow over time in the post-injury recording. The umbra and penumbra are labeled, showing increase in penumbra values over time. (**C**,**D**) Plotting the individual parameters over time shows total reduction in umbra flow with no recovery, unaffected distal tissue, and the development of the penumbras, with flow increasing during the first 5–10 min after injury. Points before 0 min are pre-injury average values.

Finally, we aimed to highlight a particular advantage of using non-contrast ultrasound over contrast-enhanced techniques: high temporal resolution of blood flow recordings. Figure 8A shows the timeline, in which we recorded blood flow continuously for 15 min pre- and post-injury. Blood flow over time in the pre-injury period can be found in Supplementary Fig. S4. Using the spatial distribution, we can plot blood flow as a function of both time and space (Fig. 8B). We see in this representative plot that flow in the umbra as well as in the healthy tissue remained constant after initial post-injury changes. However, both penumbra peaks seemed to be delayed in their formation, starting off at either lower or equal value to the pre-injury baseline.

This phenomenon was observed across all five rats in both the rostral and caudal tissue (Fig. 8C,D). The flow in the umbra was abolished immediately after injury and did not recover within the first 15 min. Flow in the healthy tissue did not significantly change at any point in the experiment. In both penumbras, however, the average flow dropped below pre-injury values at first, before climbing to a plateau 5–10 min after the injury. Additionally, the variance of each penumbra was greater than the other flows, as reflected by the error bars, indicating that this may be a more sensitive parameter to small changes in the injury and/or animal physiology.

## Discussion

In the following section, we will discuss the methodology for determining the spatial distribution of blood flow. We will follow this with an evaluation of the comparison of our non-contrast technique to contrast-enhanced ultrasound. We will further explore the blood flow distributions, with emphasis on the somewhat unexpected increase in blood flow in the penumbra. Finally, we will discuss the ability of non-contrast ultrasound to continuously measure spinal cord blood flow and subsequent clinical applications.

### Methodology to determine the spatial distribution of blood flow

The method for calculating the spatial distribution of spinal cord blood flow was chosen as a simple way to visualize the extent of injury. The inspiration for the calculation came from Soubeyrand and colleagues^16,17^, who used contrast-enhanced ultrasound to quantify blood flow. By drawing seven regions of interest distributed rostro-caudally, they showed a decrease in blood flow at the injury epicenter. We expanded upon this method through a more rigorous definition of distance from injury and by increasing the spatial resolution of the distribution through smaller distance bins. Furthermore, we have included benchtop validation of our velocity index measure (Fig. 4). In this experiment, the direction of flow of the phantom was at an angle to the transducer (Supplementary Fig. S1A). This would require a correction if directly estimating velocity from velocity index. However, since we have limited our conclusions on the velocity index measure to showing monotonicity of the relationship between velocity index and velocity, keeping the angle between the phantom and transducer constant is sufficient. Consequently, we can conclude in further figures that an increase in velocity index generally signifies an increase in flow velocity. Exact calculations of flow velocity, however, remain unattainable by this method. As such, we have described all further changes in terms of velocity index, in arbitrary units, without reference to absolute measures of velocity. Additionally, the sulcal arteries in humans range from 60 to 400 µm in diameter^47–50^, and are likely even smaller in rats. Our phantoms only encompassed the upper end of this spectrum, as the poor sonolucency of PDMS limited measurement of slow flow in smaller vessels. Further characterization using smaller microfluidic phantoms and more sonolucent polymers may help to address these limitations.

We also chose to investigate the rostral-caudal distribution, as that enabled measurement of both affected and healthy tissue. The bin size of 0.1 mm was chosen to balance increased spatial resolution with high enough sampling of pixels (on the order of 4000 pixels per bin). We have discussed the shape of the distribution in detail (see section “Effect of injury on the blood flow distribution”), but briefly, the distribution shows a central decrease in blood flow (umbra) with peripheral increase in blood flow in each direction (penumbra). It should be noted that the umbra is not always centered at exactly the 0 mm distance (Fig. 6), likely due to error introduced by manual drawing of the injury location. However, we do not believe this error is significant in magnitude to the results of the study, as it is only 2% of the overall spatial domain, and we do not draw any conclusions about the absolute injury position. In addition to the sagittal view, it may be interesting in future work to study axial images to investigate perfusion from the smaller posterior spinal arteries^31^.

### Comparison to contrast-enhanced imaging

Blood flow to tissue as measured by ultrasound can be split into two categories: flow through relatively large vessels and perfusion through capillaries and the extracellular space^26^. The main limitation of SMI is that it only images vasculature and misses capillary perfusion. As mentioned in the introduction, this means that areas that lack flow on non-contrast images may be perfused by an out-of-plane vessel, resulting in artificially low measures of blood flow. Newer non-contrast techniques, such as functional ultrasound, partially overcome this limitation through innovative beam-forming techniques and high sampling rates in the range of kHz^30^. However, in the case of the spinal cord, the layout of the main sulcal arteries within the mid-sagittal plane (Fig. 1B) allows us to overcome this problem even with a simple linear, clinically available probe. Conversely, generalizability of this analysis to other organ systems may be limited if their vasculature does not conveniently fall in a single plane. For example, the brain’s vasculature is morphologically more complex than that of the spinal cord, and the vasculature cannot be easily measured with one imaging plane^51^. To counter this, it may be useful to utilize a 3-dimensional image and distribution to map the blood flow over time (4D/real-time 3D ultrasound)^20,52^. Alternately, functional ultrasound may be able to measure capillary perfusion and may be a more viable non-contrast method to use clinically in situations with non-planar blood flow^53^.

Our results show that there is rank correlation between our contrast-enhanced and SMI images (average ρ = 0.55). Spearman’s rank correlation was used to avoid making any assumptions of linearity between the two measures. When performed on multiple rats, the average of correlations was calculated using Fisher’s *z* transformation as opposed to a single correlation calculated on data from all rats. This transformation was used as the contrast-enhanced values seemed to vary between rats more than the non-contrast values. Such variability could have been due to inconsistencies in the contrast injection technique, despite using an image generation method (i.e., time constant analysis) that was more robust to injection amount errors^22,23^. Such errors were introduced due to the need for rapid bolus injection of a small amount (400 µL) of concentrated contrast at high rate, resulting in relatively variable peak contrast concentrations. Future studies could use a different contrast technique, such as the destruction-replenishment method^17,23^ or singular value decomposition of high framerate image sequences to address this issue^26^.

Non-contrast and contrast-enhanced measurements were also performed as close to each other as possible (1–2 min). This permitted the assumption that minimal blood flow change would occur over that time scale, although this may not be true in the penumbra, as shown in Fig. 8. Notably, one of the rats showed no correlation between the two modalities (Fig. 5E), perhaps due to poor positioning of the probe resulting in the imaging plane not being optimally aligned to the midsagittal plane of the rat. Removing this rat from the analysis resulted in an average correlation of 0.67. We included this rat to show the limitations of our system and to emphasize the importance of careful probe positioning. In human patients, this may also prove to be less limiting, as larger overall vessels permit greater errors in the imaging plane. Overall, this demonstrates that careful non-contrast measurements may function as an adequate substitute for contrast-based methods, especially when contrast-based methods are unavailable or untenable, such as long-term or repeated clinical measurements.

### Effect of injury on the blood flow distribution

Using SMI images, we have consistently noted a pattern of blood flow following injury with decreased flow at the injury epicenter, or umbra. Immediately peripheral to the umbra, we see increased flow, which we define as the penumbra. Distal tissue in each direction seems relatively unaffected. Qualitatively, this concept is illustrated in Fig. 6, with statistical analysis in Fig. 7. It should be noted that the penumbra height distributions in Fig. 7B,F have large variance, indicating that penumbra hyperperfusion is not necessarily consistent. The representative rat in Fig. 5C illustrates this concept, as the rostral penumbra in this rat was not as drastically hyperperfused, especially in the contrast-enhanced distribution. However, across all rats, the pre- to post-injury pairwise comparison (Fig. 7B,D,F,H) was robust, supporting the concept that after injury, penumbra flow tends to increase in general. To further verify these results and ensure they are not due to imaging error between the pre- and post-injury timepoints, we demonstrated that flow through injury regions showed no change after probe removal and replacement (Supplementary Fig. S3).

The presence of increased flow in the penumbra areas is perhaps the most surprising phenomenon that we have noted in this study. Many of the studies that have used contrast-enhanced imaging have only shown decreased perfusion around the injury site, without peripheral hyperperfusion^12–16, 24^. The authors in most of these studies did not generate spatial distributions in the same way that we have demonstrated here, and therefore may have missed the injury zones revealed by this analysis. Moreover, Soubeyrand et al.^16^ generated a similar distribution to our study, albeit with only seven different sites through space, and showed only decreased perfusion around the injury. On the other hand, some older studies have noted increased perfusion after spinal cord injury, which has been variably attributed to low injury severity, increased blood pressure, or sampling site^18^.

Our results, with a hyperperfused penumbra, shed more light on the perfusion distribution than has been previously observed. We believe the pattern observed here may have a biological explanation. One potential interpretation is increased flow to the penumbra within larger arterioles which does not make it to the capillaries. Since there is disruption to the blood spinal cord barrier after SCI^54^, blood collects in the extracellular space and does not adequately perfuse tissue. In that case, we might expect the discrepancy between contrast-enhanced and non-contrast ultrasound to change depending on the location around the injury. Although we did observe some dependence of this discrepancy on injury zones (Supplementary Fig. S2B), it may be worthwhile to perform a more targeted study to quantify differences between flow and perfusion in the different injury zones. An ultrasound imaging technique that can simultaneously record both types of vasculature would be ideally suited for this, such as the singular value decomposition of non-linear Doppler sequences developed by Bruce et al.^12,26^.

Gallagher et al. have also defined the penumbra as an area of decreased flow in humans with the use of laser speckle contrast imaging^19,55^. However, in one of their figures, it appears that there may be a hyperperfused penumbra visible, and they also describe hyperperfusion and patchy perfusion modes of injury. Perhaps this phenomenon is due to the higher variability of SCI encountered in human patients outside the controlled lab setting. As a result, blood flow distributions may be more variable as well, and the penumbra may be less spatially defined. Our results also suggest the possibility of regions with both increased and decreased blood flow, and further study of more heterogeneous modes of injury may shed light on this phenomenon.

### Injury severity effects

Although our technique was highly effective at detecting post-injury effects when compared to pre-injury, it faced limitations in delineating the effects of injury severity (Fig. 7). Other groups have demonstrated statistically significant correlations between injury severity, measured through impact force and behavioral testing, and contrast-derived flow parameters^12,24^. We see some statistically significant differences as well, but the magnitudes of changes are not large overall. The prominence parameter (Fig. 7D,H) showed the greatest dependence on injury severity, which is expected as it is affected by changes in both the umbra and penumbra flow.

Additionally, we performed no functional or histological analyses to verify the results. Instead, we show these findings as proof-of-concept, demonstrating that this clinically available non-contrast ultrasound system can detect effects of injury force on blood flow. To make stronger conclusions on the effects of injury severity, this experiment could be repeated with behavioral and histological analysis. Some differences between the rostral and caudal penumbras were also observed. Although this phenomenon could be explained anatomically, as arteries supplying the anterior spinal vasculature enter at specific spinal levels, we believe a more targeted study is needed before drawing conclusions.

### Tracking through time

By tracking blood flow in the first 15 min after injury (Fig. 8), we have shown that umbral blood flow is annihilated after a severe SCI, distal blood flow appears relatively unaffected, and the penumbra seems to develop within the first 5–10 min after injury. One potential explanation for this may be that capillary perfusion in both the umbra and penumbra is compromised by the injury. This explanation is the consensus of the literature discussed above (see section “Effect of injury on the blood flow distribution”). In the umbra, if the larger vasculature is also compromised, flow would not be able to recover. However, in the penumbra, the decrease in perfusion, combined with blood spinal cord barrier disruption^54^, would result in a local buildup of CO_2_ causing vasodilation^32,56^. As a result, arteriolar flow, as measured by non-contrast imaging, may increase to this site. However, there would not be a corresponding perfusion increase through the compromised capillaries, as measured by contrast-enhanced ultrasound. This hypothesis may explain the difference between contrast-enhanced and non-contrast measurements, but requires further experiments to confirm.

The progression of blood flow has been captured in the first hour after injury using contrast-enhanced ultrasound^16^. The authors of that study found a general decrease in blood flow in all regions of the spinal cord in the hour after injury, although this was also seen in the uninjured group, likely indicating a non-injury cause of the decrease. Otherwise, our results agree with this study, showing relative decrease in perfusion at the epicenter when compared to healthy tissue. Again, they did not note an increase at the penumbra, further highlighting the potential difference between flow and perfusion. Furthermore, they were only able to measure flow every 5 min^57^, a far lower sampling rate than our rate of 39 Hz. Newer contrast-enhanced techniques^12,24, 26^ should potentially overcome this weakness, enabling simultaneous high temporal resolution tracking and future investigations of the difference between perfusion and flow on a temporal scale.

### Clinical translatability

The true advantage of non-contrast ultrasound lies in its ability to provide real-time monitoring of the injury site, currently unfeasible in the inpatient setting. We show here proof-of-concept of translation through a high temporal resolution 15-min recording after injury. By recording blood flow through time and distance from injury (Fig. 8B), we can separate the signal into clinically relevant regions: the umbra, penumbra, and healthy tissue (Fig. 8C,D). Unlike contrast-enhanced ultrasound, there is no clinical limitation on the length of this recording, as no agent is continuously injected into the patient. This opens the door to assessing blood flow in real-time, allowing clinicians to treat hypoperfusion based on end-organ data, thus optimizing their acute management of the injury. As mentioned in the introduction, we are currently confined to treating systemic blood pressure or spinal cord perfusion pressure as a surrogate for what is happening at the level of the spinal cord, with all its associated limitations. Our observation of different injury zones presents the opportunity for further study of how interventions affect specific areas, like the penumbra. Future studies will go further into clinical application, as we investigate interventions commonly performed in the ICU to maximize function after the acute phase of SCI. Continuous blood flow monitoring has revolutionized the care of diseases such as stroke^58^ and traumatic brain injury^59^, as both a prognostic marker and therapeutic guide, with interventions primarily aimed at recovering penumbra tissue.

Although we have demonstrated the ability of non-contrast ultrasound to record blood flow and detect dynamic changes for an unrestricted period, challenges remain before clinical implementation can be achieved. Intraoperative use of ultrasound is already possible^60–62^, and obtaining a short intraoperative recording would not be overly burdensome to the surgeon. However, this would likely be limited to ~ 15 min, and would provide only a short snapshot of the injury. To measure flow in the spinal cord transcutaneously, the transducer must have sufficient penetration depth, which results in sacrifice of spatial resolution. Several groups are working on designing implants that would rest directly over the dorsal cord, maximizing signal quality and ensuring sufficient penetration depth. Such a device would allow integration of blood flow as a variable into the intensive care unit workflow, providing real-time insight into the injury site and efficacy of therapeutic interventions.

## Conclusions

Traumatic spinal cord injury results in significant blood flow changes that may affect the course of recovery and overall patient outcomes. As a result, spinal cord blood flow imaging may be a useful clinical tool for physicians to use in the intensive care unit. Most blood flow imaging techniques are limited by either continuous contrast injection or inaccuracy of measurement. Here, we introduce the use of a clinically available ultrasound transducer for non-contrast imaging and monitoring blood flow in rat spinal cord after injury. This technique results in spatial distributions of blood flow after injury comparable to those generated using contrast-enhanced ultrasound without the limitations of a continuous infusion. Furthermore, we have shown a new blood flow pattern after injury with a decrease in flow at the injury epicenter, or umbra, and increase in flow in the immediate periphery, or penumbra. Tracking the regions through time with high temporal resolution shows the development of the penumbra in the first 10 min after injury and highlights the clinical potential. We hope that our approach will set the stage for future investigations on penumbral blood flow response and restoration. Our non-contrast imaging technique will enable blood flow monitoring as a new clinical paradigm for the acute treatment of spinal cord injury.

### Supplementary Information

Supplementary Information.

## Data Availability

All data, including images and processed files, will be made available upon reasonable request to A.M.

## Abbreviations

SCI: Spinal cord injury
SMI: Superb microvascular imaging (non-contrast imaging modality)

## Code availability

The code used to analyze the data can be found at https://github.com/HEPIUSLAB/spatial_distribution_2023.
