# Supplementary material for: Non-contrast ultrasound image analysis for spatial and temporal distribution of blood flow after spinal cord injury
Source: Sci Rep. 2024 Jan 6;14:714. doi: 10.1038/s41598-024-51281-7 (PMC10771432; doi:10.1038/s41598-024-51281-7)
Supplement: Supplementary file 1 — Supplementary Information. [file 41598_2024_51281_MOESM1_ESM.pdf]

NON-CONTRAST ULTRASOUND IMAGE ANALYSIS FOR SPATIAL AND TEMPORAL DISTRIBUTION OF BLOOD FLOW AFTER SPINAL CORD INJURY

Denis Routkevitch [a-c], Zoe Soulé [a], Nicholas Kats [a], Emily Baca [a], Andrew M. Hersh [b-c], Kelley M. Kempski-Leadingham [b,c], Arjun K. Menta [b,c], Meghana Bhimreddy [b,c], Kelly Jiang [b,c], A. Daniel Davidar [b,c], Constantin Smit [c], Nicholas Theodore [b,c], Nitish V. Thakor [a,c,d], Amir Manbachi [a-f,*]

[a]: Department of Biomedical Engineering, Johns Hopkins University, Baltimore, MD, USA

[b]: Department of Neurosurgery, School of Medicine, Johns Hopkins University, Baltimore, MD, USA

[c]: HEPIUS Innovation Laboratory, School of Medicine, Johns Hopkins University, Baltimore, MD, USA

[d]: Department of Electrical and Computer Engineering, Johns Hopkins University, Baltimore, MD, USA

[e]: Department of Mechanical Engineering, Johns Hopkins University, Baltimore, MD, USA

[f]: Department of Anesthesiology and Critical Care Medicine, School of Medicine, Johns Hopkins University, Baltimore, MD, USA

* Corresponding Author: Amir Manbachi, amir.manbachi@jhu.edu

1. <u>Supplementary Material</u>

Supplementary Table S1: Experimental design. Number of rats assigned to each experiment along with recording length. The total number of rats used was 34.

	Injury Severity and Contrast Comparison				Continuous Monitoring
**Injury Severity**	100 kDyn	175 kDyn	250 kDyn	Control	250 kDyn
**Number of rats**	10	5	9	5	5
**Pre-injury recording length (non-contrast)**	5 seconds	5 seconds	5 seconds	5 seconds	15 minutes
**Post-injury recording length (non-contrast)**	5 seconds	5 seconds	5 seconds	5 seconds	30 minutes
**Number of rats undergoing contrast injection**	2	2	3	0	0
**Post-injury contrast-enhanced recording length**	150 seconds	150 seconds	150 seconds	N/A	-

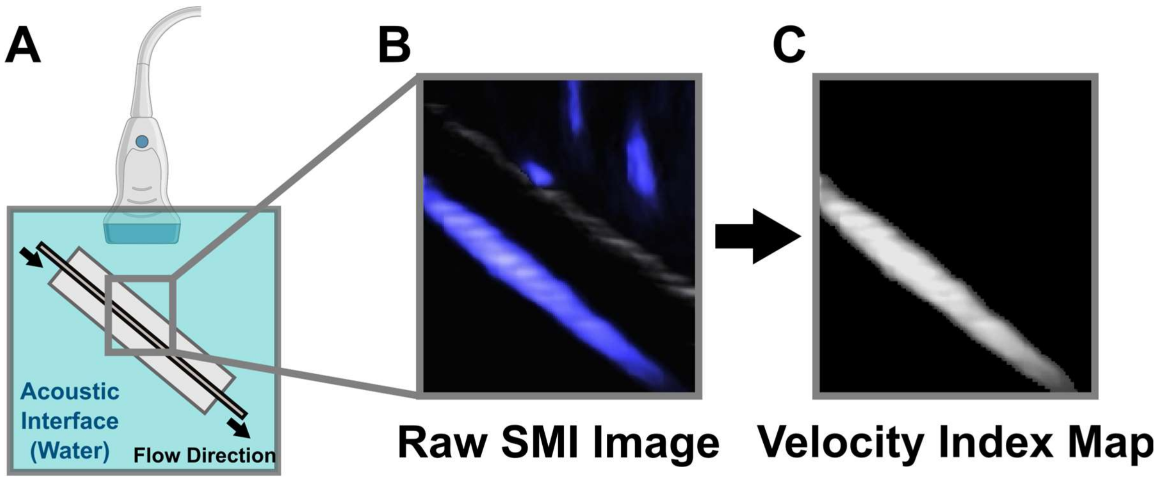

Supplementary Figure S1: Diagram and Example Images of Benchtop Experiment. (A) A diagram of the experimental procedure. The phantom was positioned in a water tank such that the direction of flow was as vertical as possible. The ultrasound transducer was placed over the phantom, with the imaging plane in line with the phantom lumen. Doppler fluid (to mimic blood) was pumped through the phantom using a syringe pump. Space restrictions prevented the direction of flow from being perfectly perpendicular to the transducer. (B) An example SMI image and (C) the processed velocity index map of the flow phantom during operation. This setup was used to generate **Figure 4**.

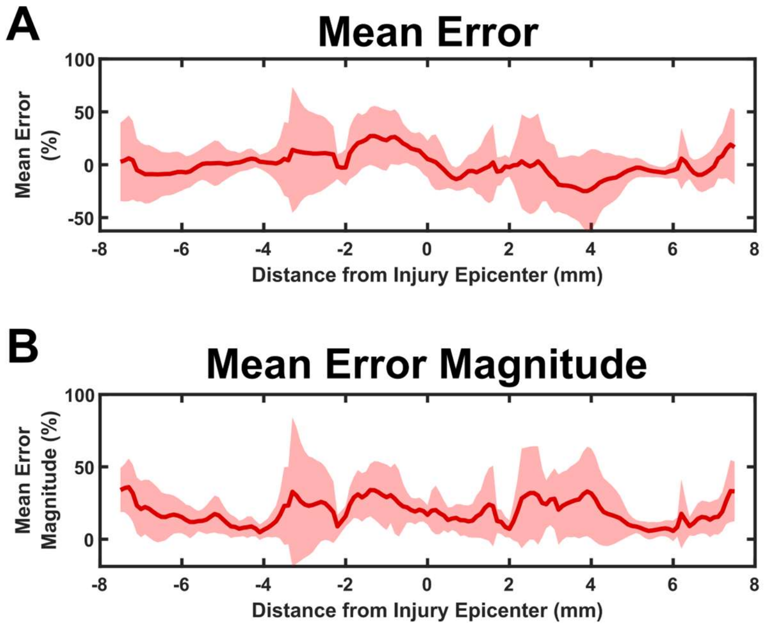

Supplementary Figure S2: Percent error between non-contrast and contrast-enhanced ultrasound as a function of distance from injury. To calculate percent error between contrast- enhanced and non-contrast modalities, non-contrast data was transformed using the spline models in **Figure 5**. Neither mean error (A) nor error magnitude (absolute value, B) show a strong relationship with distance from injury. There may be slight increase in error magnitude at either side of the injury (0.5-4 mm distance in both directions), that could be attributed to differences in the recording modalities at penumbra locations. Error was calculated from scaling curves by their mean using the data depicted in **Figure 5**.

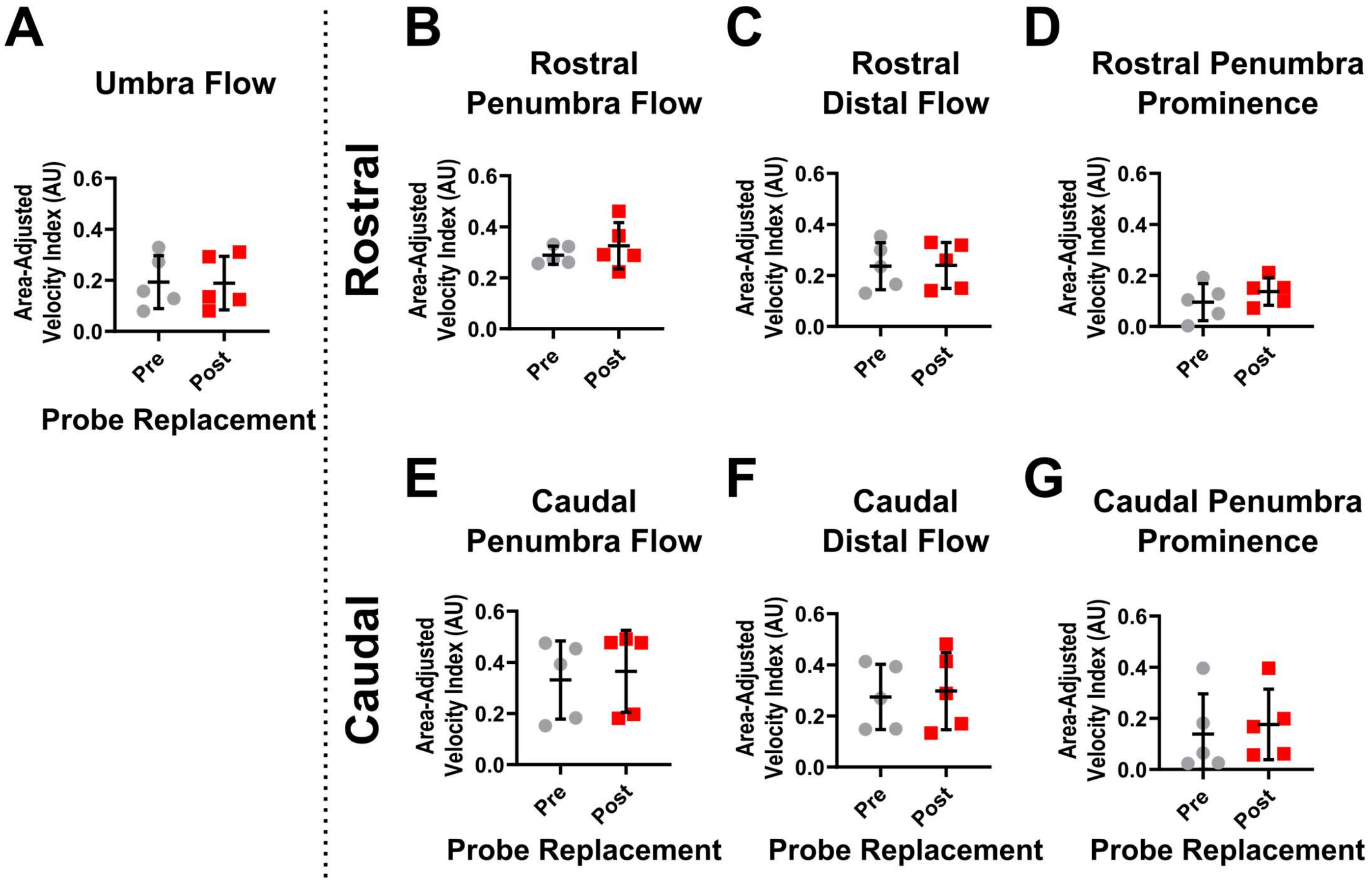

Supplementary Figure S3: Spatial distribution parameters as calculated from probe removal and replacement show no significant change in parameter values. To verify that probe replacement was not a significant factor in the results shown in **Figure 7**, spatial distribution parameters were calculated pre- and post-probe replacement. The “injury site” was labeled as a line perpendicular to the spinal cord approximately in the center of the image. No significant change was seen in umbra flow (A), penumbra flow rostral (B) and caudal (E), distal flow rostral (C) and caudal (F), and in the penumbra prominence rostral (D) and caudal (G). Injury extent not shown as there was no site of injury. None of the comparisons achieved statistical significance.

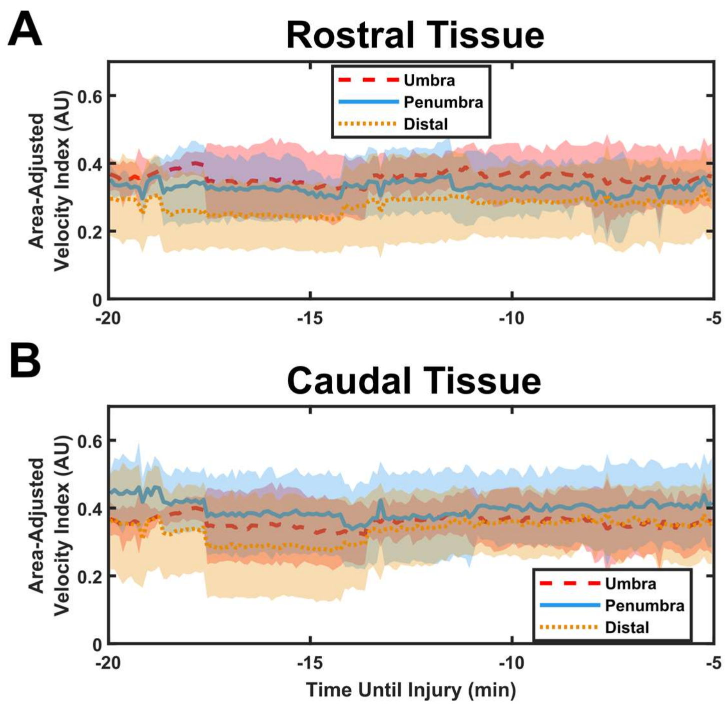

Supplementary Figure S4: Time Series of Pre-Injury Recordings. Time series recordings of each injury region for rostral (A) and caudal (B) regions shows constant flow in the 15-minute pre-injury recordings. The data are shown in condensed format in **Figure 8C,D**.
